# Determining novel functions of *Arabidopsis *14-3-3 proteins in central metabolic processes

**DOI:** 10.1186/1752-0509-5-192

**Published:** 2011-11-21

**Authors:** Celine Diaz, Miyako Kusano, Ronan Sulpice, Mitsutaka Araki, Henning Redestig, Kazuki Saito, Mark Stitt, Ryoung Shin

**Affiliations:** 1 RIKEN Plant Science Center, Yokohama, Kanagawa 230-0045, Japan; 2 Max Planck Institute for Molecular Plant Physiology, Potsdam-Gölm, 14476, Germany

## Abstract

**Background:**

14-3-3 proteins are considered master regulators of many signal transduction cascades in eukaryotes. In plants, 14-3-3 proteins have major roles as regulators of nitrogen and carbon metabolism, conclusions based on the studies of a few specific 14-3-3 targets.

**Results:**

In this study, extensive novel roles of 14-3-3 proteins in plant metabolism were determined through combining the parallel analyses of metabolites and enzyme activities in 14-3-3 overexpression and knockout plants with studies of protein-protein interactions. Decreases in the levels of sugars and nitrogen-containing-compounds and in the activities of known 14-3-3-interacting-enzymes were observed in 14-3-3 overexpression plants. Plants overexpressing 14-3-3 proteins also contained decreased levels of malate and citrate, which are intermediate compounds of the tricarboxylic acid (TCA) cycle. These modifications were related to the reduced activities of isocitrate dehydrogenase and malate dehydrogenase, which are key enzymes of TCA cycle. In addition, we demonstrated that 14-3-3 proteins interacted with one isocitrate dehydrogenase and two malate dehydrogenases. There were also changes in the levels of aromatic compounds and the activities of shikimate dehydrogenase, which participates in the biosynthesis of aromatic compounds.

**Conclusion:**

Taken together, our findings indicate that 14-3-3 proteins play roles as crucial tuners of multiple primary metabolic processes including TCA cycle and the shikimate pathway.

## Background

14-3-3 proteins are known to regulate diverse processes via binding phosphorylated target proteins in all eukaryotes [1-5]. Although hundreds of potential 14-3-3-interacting proteins have been identified [1,5], there have been limited studies that confirm *in vivo *interactions and/or elucidate the regulating functions of 14-3-3 proteins [6-10]. The most intensively characterized 14-3-3 target proteins are nitrate reductase and H^+^-ATPase. 14-3-3 proteins activate H^+^-ATPase [11] and inhibit nitrate reductase activity [12]. Our previous study suggests that three 14-3-3 isoforms (kappa, chi and psi) also play important roles in nitrogen and sulfur metabolic processes by regulating the activities of phosphoenolpyruvate carboxylase and O-acetylserine lyase [13].

Plant 14-3-3 proteins are mainly thought to be regulators of carbon and nitrogen metabolism [2]. However, this assumption is based on studies of only a few target proteins, such as nitrate reductase and sucrose-phosphate synthase [14]. Nitrate reductase is phosphorylated in the dark by the calcium-dependent protein kinase (CDPK) and the sucrose non-fermenting related kinase 1 (SnRK1) that initiates the interaction of the enzyme with the 14-3-3 proteins and its inactivation. In the light, nitrate reductase is dephosphorylated by a protein phosphatase 2A, leading to the dissociation of the 14-3-3 and the activation of nitrate reductase [15-18]. In carbon metabolism, some carbon metabolic enzymes such as sucrose phosphate synthase [19], and the dual function protein 6-phosphofructo-2-kinase/fructose-2,6-bisphosphatase [20], have been identified as interacting targets of 14-3-3 proteins. The functional relevance of 14-3-3 proteins in the regulatory mechanism of their targets, however, is still not clear. Considering the hundreds of possible 14-3-3 target proteins revealed through multiple screening studies, the roles so far described are likely to be only a small part of the functions of 14-3-3 proteins [5,13,21].

Metabolite profiling is a powerful tool that has contributed to the understanding of plant physiology, including phenotypic differences, gene annotations, metabolite regulation, and characterization of stress responses [22,23]. Moreover, the integration of metabolomics with other 'omics,' such as genomics, enzymomics, and interactomics, leads not only to construction of metabolic networks but also to understanding the roles particular proteins play within the metabolic network [24,25]. In this study, by combining metabolomics and genetical, enzymological, biochemical, and molecular approaches, we were able to draw a comprehensive map of the functional roles 14-3-3 proteins play in essential metabolic processes.

Our study further confirms that 14-3-3 proteins are important regulators of both nitrogen and carbon metabolic processes. Specifically, we show that 14-3-3 proteins play roles to control the tricarboxylic acid (TCA) cycle and the shikimate pathway.

## Results

### Metabolite profiling: ectopic expression of 14-3-3 proteins altered primary metabolite levels

Our previous studies demonstrated that 14-3-3 chi, kappa and psi proteins interact with more than a hundred proteins and that these interactions regulate the activities of some metabolic enzymes [13]. However, it remained unclear why 14-3-3 proteins interact with so many proteins and what their true targets are *in planta*. To comprehend the biological roles of 14-3-3 proteins, metabolic profiling was performed on plants overexpressing a 14-3-3 protein (14-3-3 ox) and on previously confirmed 14-3-3 kappa knockout plants (kappa-KO), 14-3-3 chi knockout plants (chi-KO) and 14-3-3 psi RNAi plants (psi-RNAi) that showed 70% reduction of endogenous 14-3-3 psi expression [13]. 14-3-3 ox plants with 14-3-3 contents of at least two times more than wild type *in planta *were used [13]. Long day (16 h light/8 h dark) plate-grown plants were divided into shoots and roots, and changes in their levels of primary metabolites relative to wild type plants were determined using GC-TOF-MS (gas chromatography-time of flight-mass spectrometry).

**Figure 1:**
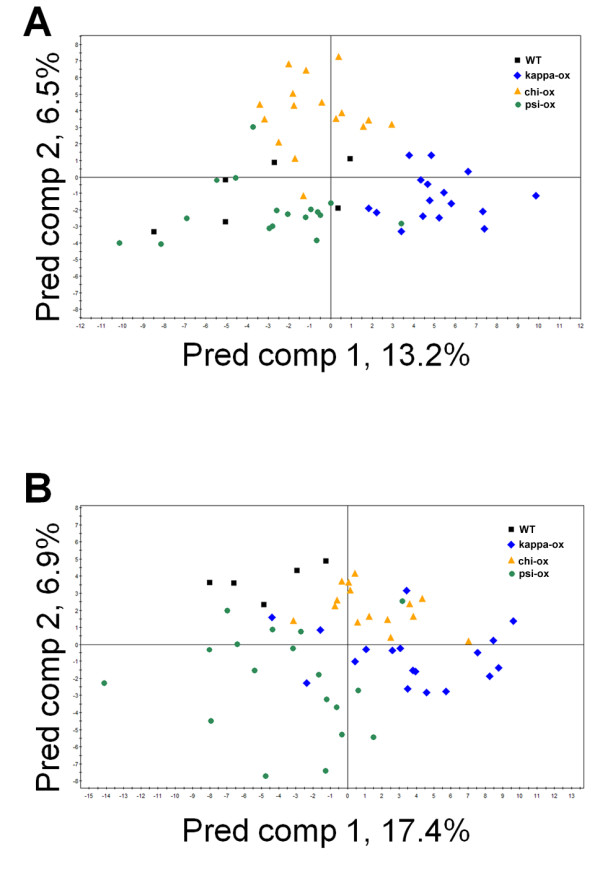
**The OPLS-DA score scatter plots of three 14-3-3 overexpressing and wild type samples for (A) shoots and (B) roots**. Each point represents an independent plant sample in the score scatter plots. We used 55 shoots and 56 roots for the analysis. (A) The OPLS-DA model for shoot samples shows three significant components, with *R^2^X*, *R^2^Y *and *Q^2^Y *values of 0.37, 0.67 and 0.41, respectively. (B) The OPLS-DA model for root samples shows three significant components, with *R^2^X*, *R^2^Y *and *Q^2^Y *values of 0.30, 0.50 and 0.23, respectively. Black square, wild type; blue diamond, kappa-ox; yellow triangle, chi-ox; green circle, psi-ox.

**Figure 2:**
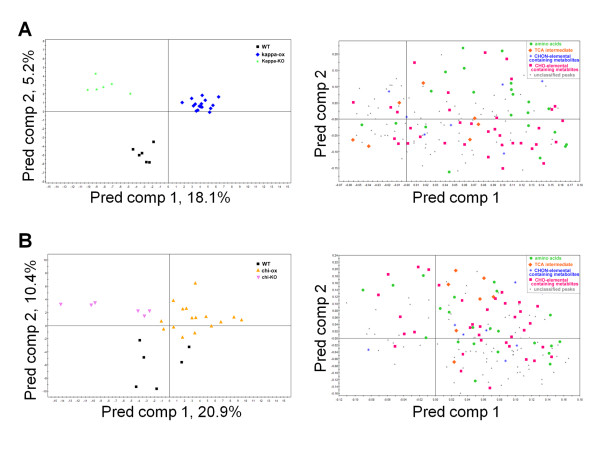
**The OPLS-DA score scatter plots (left) and loading scatter plots (right) of shoot samples of (A) kappa-ox and kappa-KO (B) chi-ox and chi-KO**. Wild type samples were used as controls. Each point represents an independent plant in the score scatter plots and an individual metabolite peak in the loading plots. (A) The OPLS-DA model of kappa samples shows two significant components, with *R^2^X*, *R^2^Y *and *Q^2^Y *values of 0.53, 0.95 and 0.65, respectively. (B) The OPLS-DA model of chi samples shows three significant components, with *R^2^X*, *R^2^Y *and *Q^2^Y *values of 0.31, 0.67 and 0.41, respectively. These models were validated using analysis of variance of cross-validated predictive residuals (CV-ANOVA) (p_CV _< 0.01). (Left) Black square, wild type; blue diamond, kappa-ox; pale-green star, kappa-KO; yellow triangle, chi-ox; green circle, psi-ox; pink-inverted triangle, chi-ox. (Right) Pale-green circle, amino acids; orange diamond, TCA intermediates; blue star, metabolites that consists of CHON-elemental composition; pink square, metabolites that consists of CHO-elemental composition; gray triangle, unclassified peaks. Number of biological replicates: wild type, n = 6; kappa-ox, n = 16; kappa-KO, n = 6; chi-ox, n = 16; chi-KO, n = 6; psi-ox, n = 17; psi-RNAi, n = 6. p_CV_, p-value of the probability level of the F-test in each model.

To visualize the metabolomic changes in 14-3-3 ox and KO plants, principal component analysis (PCA) was conducted using metabolite profile data matrix to plot the samples' distribution. 14-3-3 kappa-ox, chi-ox, and psi-ox were distributed in distinguishable clusters, with kappa-ox having the most significantly different metabolite profile compared to wild type (Figure 1). The subsequent supervised method, orthogonal projections to latent structures-discriminant analysis (OPLS-DA) reconfirmed that 14-3-3 ox plants have different metabolic profiling patterns compared to wild type (Figure 1). The scatter plot showed that the metabolites, such as amino acids, TCA intermediate and carbohydrates, of 14-3-3 kappa-ox were clearly distinguishable from that of 14-3-3 kappa-KO and wild type (Figure 2A); and the chi-ox plants showed similar trends of metabolites distribution as 14-3-3 kappa-ox (Figure 2B).

The 14-3-3 ox lines and KO lines had clear alterations of many metabolites (Table 1 and Additional file 1). Twelve metabolite contents were significantly modified in the roots; and twenty five, in the shoots (Table 1). 14-3-3 chi-ox and 14-3-3 kappa-ox roots had decreased levels of some amino acids, such as alanine, phenylalanine and glutamate, compared to wild type but fewer metabolite changes were found in the roots of 14-3-3 psi-ox and KO plants for all three 14-3-3 proteins. Interestingly, the changes of metabolites in the shoots were more pronounced-the levels of many metabolites that decreased in 14-3-3 ox shoots increased in KO shoots (Table 1). Metabolites differentially regulated in 14-3-3 ox shoots were divisible into four groups. First, many amino acids, including alanine, glycine, and lysine, decreased in 14-3-3 ox plants. Second, sugar levels decreased in 14-3-3 ox lines. Third, metabolite contents of TCA cycle, such as malate and citrate, decreased in 14-3-3 ox plants. However, levels of α-ketoglutarate increased in kappa-ox but decreased in psi-ox. Fourth, metabolites related to the shikimate pathway were altered. The contents of phenylalanine decreased in 14-3-3 kappa-, chi- and psi- ox lines. In addition, tyrosine amount decreased in kappa ox plants and the contents of shikimate increased in kappa KO plants (Table 1). From these results, it can be hypothesized that 14-3-3 proteins are involved in the regulation of TCA cycle, sugar metabolism and the shikimate pathway.

**Table 1:** List of signature metabolites that were changed in 14-3-3 overexpression plants compared to wild type plants.


		**Fold change compared to wild type plants**											
		
	**Metabolite**	**kappa-ox1**	**kappa-ox2**	**kappa-ox3**	**kappa-KO**	**chi-ox1**	**chi-ox2**	**chi-ox3**	**chi-KO**	**psi-ox1**	**psi-ox2**	**psi-ox3**	**psi-RNAi**

Shoot	β-alanine	**0.613**	**0.741**	**0.628**	**1.401**	0.809	0.912	1.002	**1.642**	**1.662**	1.296	0.999	2.087
	
	GABA	**0.682**	**0.527**	**0.440**	1.271	**0.627**	0.886	**0.647**	0.836	1.101	**0.571**	**0.578**	0.758
	
	threonic acid	**0.616**	**0.657**	**0.641**	1.178	1.106	1.324	0.859	0.945	0.973	**0.662**	0.869	**1.524**
	
	phenylalanine	**0.746**	**0.764**	**0.572**	1.050	**0.702**	**0.798**	1.032	1.009	1.102	**0.813**	**0.692**	**0.793**
	
	1,3-diaminopropane dihydrochloride	**0.546**	**0.496**	**0.413**	0.875	**0.439**	**0.485**	**0.478**	0.711	0.761	**0.574**	**0.473**	**0.552**
	
	ribose	**0.747**	**0.618**	**0.415**	**1.501**	**0.651**	0.929	0.809	1.110	1.358	0.876	**0.740**	1.102
	
	citrate	0.825	**0.735**	**0.733**	1.154	**0.444**	**0.654**	**0.538**	0.799	1.222	**0.595**	**0.670**	**0.712**
	
	fructose	**0.439**	**0.447**	**0.479**	0.771	**0.335**	**0.419**	**0.426**	0.904	**0.670**	**0.500**	**0.461**	0.914
	
	tyrosine	**0.639**	**0.684**	**0.323**	0.681	1.013	1.107	1.082	0.991	**1.687**	1.036	0.969	1.130
	
	glycine	**0.448**	**0.402**	**0.364**	0.984	**0.517**	**0.485**	**0.722**	0.786	**1.384**	0.935	**0.640**	**0.704**
	
	aspartate	0.860	**0.707**	**0.648**	**1.936**	**0.764**	1.115	1.068	**1.535**	**1.258**	1.027	0.926	**1.181**
	
	pyroglutamate	0.855	**0.833**	**0.590**	**1.709**	**0.775**	1.008	1.041	**1.444**	1.051	0.873	**0.758**	0.869
	
	glutamate	0.921	1.044	**0.766**	**1.734**	0.850	1.089	0.934	**1.375**	1.132	1.046	0.935	1.083
	
	asparagine	0.922	**0.561**	**0.350**	**2.059**	0.762	1.338	0.979	1.393	1.351	0.917	0.710	1.363
	
	glutamine	0.697	**0.439**	**0.214**	0.858	0.696	0.986	0.703	**0.578**	1.484	1.213	0.743	1.283
	
	glucose	**0.451**	**0.387**	**0.278**	0.723	**0.294**	**0.335**	**0.336**	0.530	**0.567**	**0.475**	**0.360**	0.720
	
	lysine	0.892	**0.687**	**0.374**	1.052	0.919	1.207	1.041	1.069	1.069	0.908	**0.729**	1.016
	
	sucrose	**0.550**	**0.454**	**0.354**	0.647	**0.489**	0.721	**0.603**	0.820	**0.501**	**0.550**	**0.251**	0.681
	
	palmitate	**0.813**	1.013	0.984	**1.193**	0.878	0.922	1.069	**1.231**	0.986	0.919	**0.804**	0.904
	
	shikimate	0.846	**0.722**	**0.602**	**1.801**	0.788	**0.776**	**0.678**	**1.322**	1.073	0.965	0.718	0.950
	
	1,4-diaminobutane	**0.609**	**0.561**	**0.484**	0.953	**1.333**	**1.522**	0.986	0.973	0.912	**0.775**	0.917	**1.758**
	
	Fructose-6-phosphate	1.156	0.800	**0.678**	**1.655**	**0.642**	0.879	0.942	1.108	1.089	0.967	**0.771**	1.071
	
	malate	**0.745**	**0.665**	**0.642**	1.017	**0.674**	0.866	**0.809**	0.847	**1.452**	0.911	0.955	1.008
	
	α-ketoglutarate	**0.566**	0.991	**0.546**	0.653	**0.465**	0.701	0.657	**0.583**	**3.061**	**1.869**	**2.120**	1.111
	
	myo-inositol	**0.746**	**0.969**	**0.562**	**1.487**	1.251	1.039	0.805	1.275	0.971	0.933	0.865	1.181

Root	β-alanine	**0.662**	0.830	**0.647**	1.331	**0.649**	0.617	**0.703**	0.891	1.009	0.621	0.749	1.153
	
	phenylalanine	**0.739**	**0.742**	**0.509**	1.314	**0.697**	**0.792**	0.842	1.158	0.964	0.849	**0.722**	0.880
	
	proline	**2.282**	1.578	0.762	1.301	**2.046**	1.700	1.254	1.908	**2.318**	1.678	**3.929**	**2.340**
	
	pyroglutamate	**0.755**	**0.734**	**0.525**	**1.341**	**0.751**	0.836	0.852	**1.250**	0.923	**0.775**	**0.529**	0.801
	
	glutamate	**0.685**	0.875	**0.546**	1.237	**0.741**	**0.766**	0.788	1.077	1.055	**0.815**	**0.762**	0.964
	
	trans-Sinapate	**0.447**	0.751	**0.409**	0.697	0.616	**0.553**	**0.516**	0.567	0.643	0.690	0.470	0.723
	
	palmitate	**0.742**	0.945	**0.814**	1.103	**0.800**	**0.784**	0.864	1.060	0.992	0.912	**0.723**	**0.845**
	
	1,4-diaminobutane	0.960	0.935	**0.767**	**1.645**	**0.635**	**0.736**	0.857	0.985	1.112	0.794	0.933	1.209
	
	Fructose-6-phosphate	**0.780**	**0.751**	**0.538**	1.251	**0.780**	0.860	0.790	1.059	0.906	0.779	**0.598**	0.908
	
	shikimate	1.946	1.912	1.702	**3.127**	1.553	1.666	1.811	1.979	**2.161**	1.733	1.909	**2.311**
	
	myo-inositol	**0.716**	0.806	**0.626**	**1.385**	0.909	**0.763**	0.821	1.249	0.980	0.867	**0.699**	1.043
	
	phytol	**0.179**	0.438	**0.141**	1.137	**0.233**	**0.154**	**0.219**	1.089	**0.374**	0.418	**0.167**	0.266

Bold letter indicates statistically significant in metabolite level in the line compared to wild type (P < 0.05).

**Figure 3:**
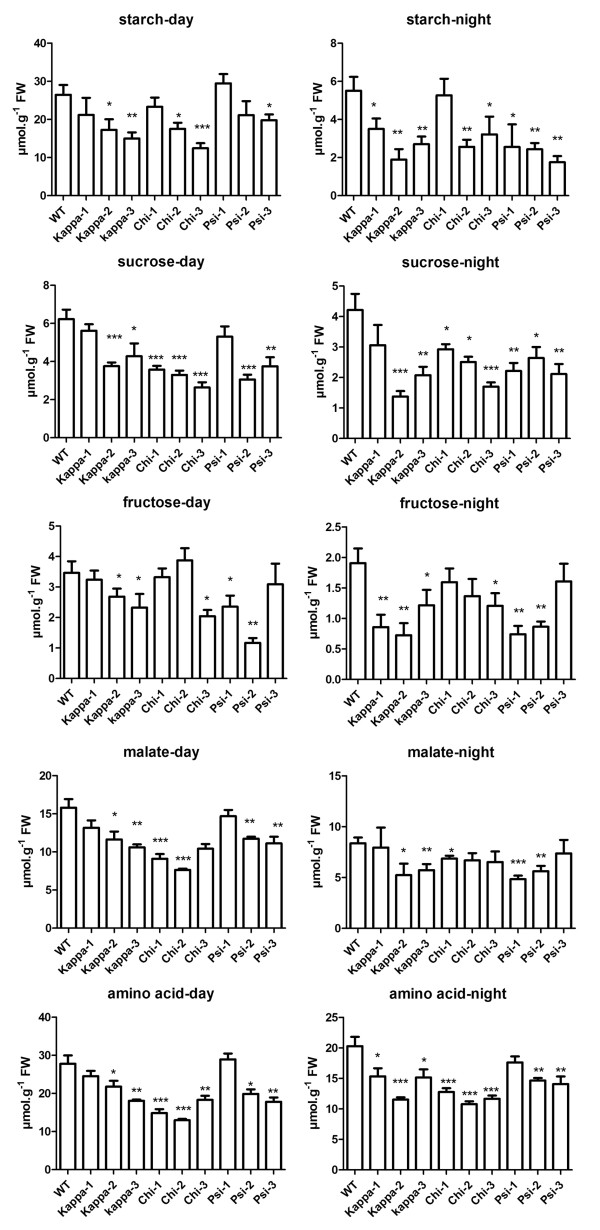
**Major metabolite changes in 14-3-3 overexpression plants during day and night**. The levels of starch, sucrose, fructose, malate and amino acids were significantly decreased in 14-3-3 overexpression plants compared to wild type (WT). Plants were harvested 1 h before switching light conditions. T-tests were performed to determine significant difference compared to WT (*, P < 0.05; **, P < 0.01; ***, P < 0.001).

It has been reported that 14-3-3 proteins and their targets are regulated by light [26,27]. Therefore, comparisons in the levels of metabolites of the 14-3-3 ox lines with that of wild type were made in light and dark conditions (Figure 3). Light and dark did not affect the overall metabolic trends in the 14-3-3 ox plants. However, starch content decrease (with the exception of chi-ox1 and chi-ox3) was more significant in the dark, at least 12% more than light; whereas malate content decrease was more significant in the light, at least 10% more than dark. The alteration of metabolite levels in 14-3-3-ox plants was therefore independent of the presence or absence of light. This indicates that the targets of the 14-3-3 proteins do not act in an exclusively light-dependent manner.

### Activity levels of primary metabolic enzymes in 14-3-3 ox plants

**Figure 4:**
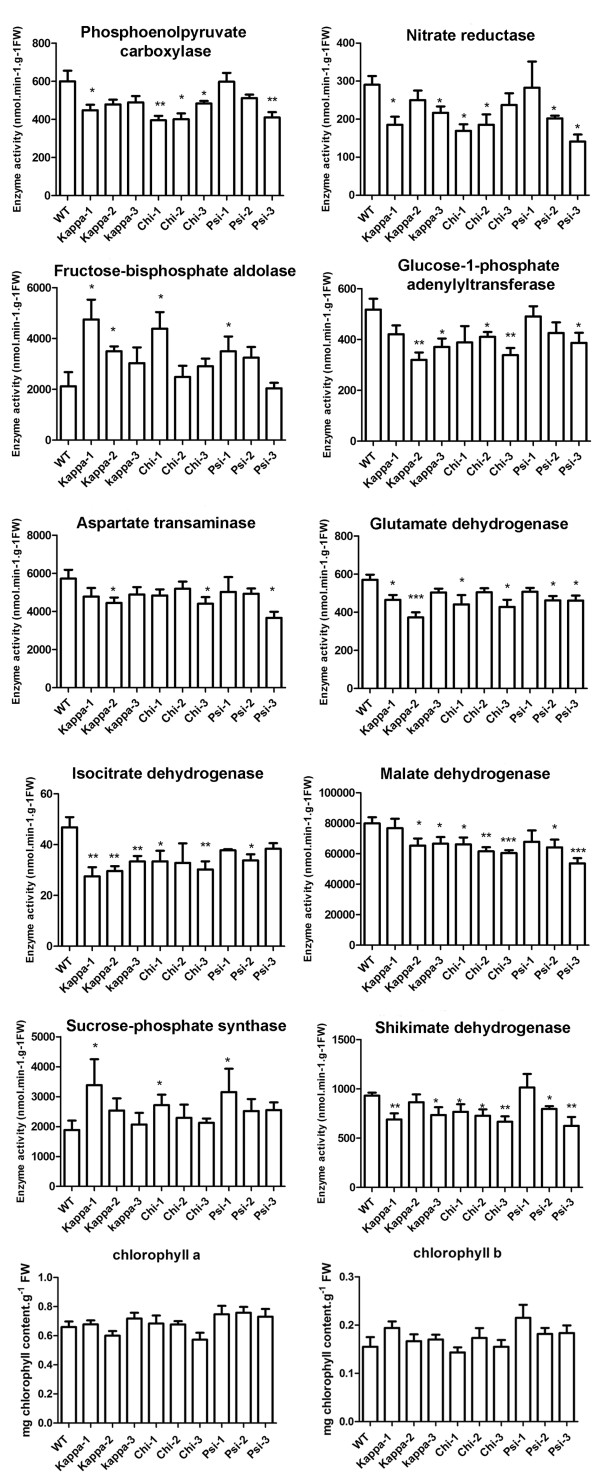
**The activities of metabolic enzymes were altered in 14-3-3 overexpression plants**. Enzyme activities were determined in 14-3-3 overexpression plants and wild type Col-0 plants (WT). Asterisks indicate significant differences compared to WT as determined by t-test with n > 7 (*, P < 0.05; **, P < 0.01; ***, P < 0.001).

The metabolite profile of 14-3-3 ox plants suggests that starch and sugar metabolism, TCA cycle, and the shikimate pathway are the main target processes of 14-3-3 proteins. To elucidate whether the enzymes of these metabolic processes are regulated by 14-3-3 proteins, the activity of 29 enzymes in 14-3-3 ox plants and wild type plants were analyzed (Figure 4 and Additional file 2). Because the most marked metabolic changes were found in the shoots (Table 1), only shoots were used for determining the enzyme activities.

We consider that there was no general reformatting of photosynthesis in 14-3-3 ox plants basing on the fact that: 1) the chlorophyll content in 14-3-3 ox plants and wild type plants were not significantly different (Figure 4); 2) the activities of 19 enzymes, including phosphoglucomutase, UDP glucopyrophosphorylase, the sucrose metabolizing enzymes glucokinase, fructokinase and β-fructofuranosidase, and the Calvin-Benson cycle enzymes triose phosphate isomerase, glyceraldehyde-3-phosphate dehydrogenase and RubisCO were not altered in the 14-3-3 ox plants (Additional file 2).

As already known from other studies [15,17,18,28], the activity of nitrate reductase decreased and the activity of sucrose phosphate synthase increased in 14-3-3 ox plants (Figure 4). Our previous findings showing that the activity of phosphoenol pyruvate carboxylase decrease and that the activity of glutamate synthase does not change in 14-3-3 ox plants [13] were confirmed in this study (Figure 4). Interestingly, several enzymes involved in TCA cycle (malate dehydrogenase and isocitrate dehydrogenase), or closely related to TCA cycle (glutamate dehydrogenase and aspartate aminotransferase) displayed decreased activities in 14-3-3 ox plants (Figure 4). The activity of citrate synthase also decreased in some of the 14-3-3 ox lines (Additional file 2). In addition to enzymes of TCA cycle, fructose bisphosphate aldolase activity increased in 14-3-3 ox plants compared to wild type plants. The decreased activity of glucose-1-phosphate adenylytransferse (Figure 4), which is involved in starch synthesis, may be responsible for the decreased starch synthesis observed in 14-3-3 ox lines (Figure 3).

### 14-3-3 proteins function in the shikimate pathway

Shikimate levels significantly increased in kappa-KO compared with wild type plants (Table 1). In addition, the enzyme activity of shikimate dehydrogenase was significantly reduced in 14-3-3 ox plants (Figure 4). Because shikimate dehydrogenase is essential for the biosynthesis of aromatic compounds in plants [29], this result is in accordance with the reduced levels of phenylalanine in the chi, kappa, and psi ox lines, of tyrosine in the kappa-ox lines, and of phytol in the 14-3-3 ox roots (Table 1). Together, these results suggest that 14-3-3 proteins play important roles in the regulation of the shikimate pathway.

### TCA cycle regulated by 14-3-3 proteins through protein-protein interaction

**Figure 5:**
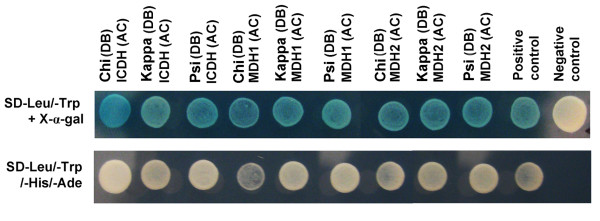
**14-3-3 proteins interact with TCA cycle enzymes in yeast**. Three 14-3-3 isoforms interact with isocitrate dehydrogenase (ICDH, At4G35650) and two malate dehydrogenases (MDH1, At1G04410; MDH2, At5G43330) that were previously isolated as possible targets of 14-3-3 proteins. AC indicates a pGADT7 and BD indicates a pGBKT7 vector. The positive control and negative control were described in methods section.

**Figure 6:**
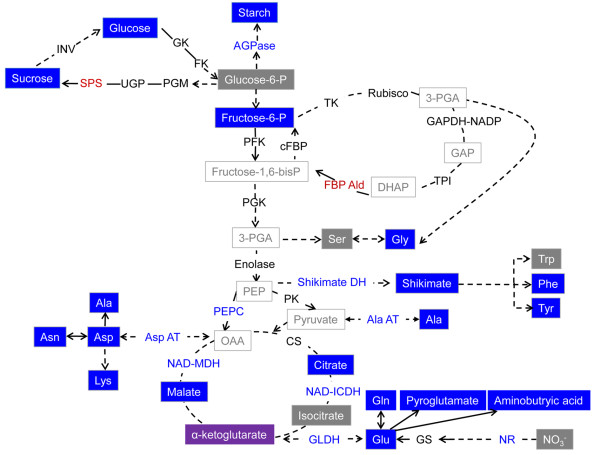
**The schematic model of metabolic pathways that are regulated by 14-3-3 proteins**. Grey box indicates unchanged metabolites; blue box indicates decreased metabolites in 14-3-3 overepxression plants compared to wild type plants; no colored box indicates that metabolites were not measured in this study; purple box (α-ketoglutarate) indicates that some overexpression lines showed higher level of metabolite compared to WT but others showed lower than WT. Black letters indicate that the activities of enzymes were unchanged in 14-3-3 overexpression plants compared to WT; Blue letters indicate that the enzyme's activity was decreased in 14-3-3 overexpression plants; Red letters indicate that the enzyme's activity was increased in 14-3-3 overexpression plants. AGPase, Glucose-1-phosphate adenylyltransferase; INV, β-fructofuranosidase; SPS, sucrose-phosphate synthase; UGP, UTP-glucose-1-phosphate uridylyltransferase; PGM, Phosphoglucomutase; PFK, 6-phosphofructokinase; cFBP, Fructose-bisphosphatase; TK, Transketolase; GAPDH-NADP, Glyceraldehyde-3-phosphate dehydrogenase (NADP^+^) (phosphorylating); TPI, Triose-phosphate isomerase; FBP Ald, Fructose-bisphosphate aldolase; PFK, 6-phosphofructokinase; PEPC, Phosphoenolpyruvate carboxylase; PK, Pyruvate kinase; Shikimate DH, Shikimate dehydrogenase; Ala AT, Alanine-glyoxylate transaminase; CS, Citrate synthase; NAD-ICDH, Isocitrate dehydrogenase (NAD^+^); NAD-MDH, Malate dehydrogenase; GLDH, Glutamate dehydrogenase; NR, Nitrate reductase; ASP AT, Aspartate transaminase.

The metabolomic profiling (Table 1 and Figure 3) and the analysis of enzyme activities in 14-3-3 ox and wild type plants (Figure 4) suggest that TCA cycle is regulated by 14-3-3 proteins. Our previous list of proteins found to interact with 14-3-3 proteins [13] includes two malate dehydrogenases (At1G04410 and At5G43330), an isocitrate dehydrogenase (At4G35650), and also an aspartate aminotransferase (At2G30970), which altered activities in 14-3-3 ox plants. To confirm whether these TCA cycle enzymes are direct targets of 14-3-3 proteins, yeast two-hybrid interaction assays were performed (Figure 5). The malate dehydrogenases and isocitrate dehydrogenase clearly interacted with 14-3-3 kappa, chi, and psi in yeast (Figure 5), suggesting that 14-3-3 proteins control TCA cycle through interaction with its metabolic enzymes, malate dehydrogenase and isocitrate dehydrogenase (Figure 6).

## Discussion

Numerous studies have shown that there are more than a hundred potential 14-3-3 target proteins in plants [3-5,30-34]. *In vivo*, the cellular distribution of 14-3-3 proteins are altered depending on the interactions with cellular clients [35]. There are numerous examples of 14-3-3 proteins interacting with and regulating various target proteins in different subcellular compartments including cytosol, nucleus, chloroplast as well as mitochondria [36-40]. These results suggest that 14-3-3 proteins localize in various subcellular compartments and play diverse roles in many cellular processes. To better understand the multi-faceted roles of 14-3-3 proteins *in planta*, a combination of metabolomics, enzyme activity analysis, and protein-protein interaction analysis was used in this study.

The interaction between nitrate reductase and 14-3-3 proteins was demonstrated in various plant species using multiple methods [15,16]. However, the decrease in nitrate reductase activity upon interaction with 14-3-3 proteins has only been measured *in vitro*, and the direct influence of 14-3-3 proteins on nitrogen metabolite levels *in planta *has not been clearly reported [16]. Through metabolomics, we found decreased levels of nitrogen containing metabolites, such as glycine, GABA, glutamine and asparagine (Table 1) possibly resulting from the alteration of nitrogen metabolic enzymes by 14-3-3 proteins (Figure 4) [41,42].

In addition to the regulation of nitrate reductase, 14-3-3 proteins control nitrogen metabolism through interaction with glutamine synthetase (GS) enzyme [8]. In *Medicago truncatula*, degradation of GS2 by proteolysis was related to the binding of 14-3-3 proteins to phosphorylated GS2 [43]. In contrast, in *Brassica napus*, 14-3-3 proteins were shown to positively regulate activity and to negatively regulate degradation of the cytosolic isoform of GS1 [38,43]. In healthy plants, the plastid-localized GS2 isoform is predominant compared to GS1 isoform. However, during senescence, GS2 degrades with the chloroplast and GS1 becomes the predominant isoform in leaves [44]. In this study, as well as in the previous study using 14-3-3 ox lines ([13]), no significant changes of GS activities were detected (Additional file 2). There are several possible explanations for the unchanged activity of GS in our conditions: 1) the change of GS activity in 14-3-3 ox plants was not great enough to be detected by our method; 2) the amount of GS proteins in 14-3-3 ox plants was a limiting factor and/or there was enough 14-3-3 protein in wild type plants to saturate GS activity; 3) alterations of metabolites related to nitrogen metabolism were not due to an alteration of GS activity but a disruption of the carbon-nitrogen balance, since drastic changes in soluble sugar and starch levels were observed in 14-3-3 ox lines (Table 1) [45-47]; 4) the method used to measure total GS activity in this study was unable to discriminate GS1 and GS2, or distinguish whether the balance between GS1 and GS2 had been modified in the 14-3-3 ox plants.

As photoperiodism is associated with drastic gene expressions, enzyme activities and metabolite level changes, samples were analyzed at time points most representative of these two periods: one hour before onset of dark when plants have accumulated maximum photosynthate such as starch, and one hour before onset of light when they have remobilized photosynthate to ensure normal growth and development. Since metabolic profiles at these two time points did not change overall metabolic trend, we can exclude photoperiodism as a factor affecting the reduction of carbohydrates in 14-3-3 ox plants.

The reduction of starch, sucrose and glucose levels in 14-3-3 ox plants (Table 1 and Figure 3) indicates that 14-3-3 proteins regulate activities of enzymes related to carbohydrate metabolism. 14-3-3 proteins bind to several enzymes of carbohydrate metabolism, such as sucrose phosphate synthase, trehalose-6-phosphate synthase and 6-phosphofructo-2-kinase/fructose-2,6-bisphosphatase [19,20,48]. Our results suggest that overexpression of 14-3-3 proteins *in planta *is associated with the modification of these carbohydrate metabolic enzymes and the decrease of sucrose and starch levels in leaves. In addition, sucrose phosphate synthase has several putative phosphorylation sites which regulate its activities by interacting with 14-3-3 proteins [8,19]. Although effect by other levels of regulation such as feedback control cannot be ignored, we hypothesize that change in soluble sugar levels in 14-3-3 ox plants resulted from the regulation of carbohydrate metabolic enzymes by 14-3-3 proteins and the lower fluxes in TCA cycle.

In this study, detailed metabolomics analysis clearly show that overexpression of 14-3-3 proteins is associated with drastic changes in the levels of TCA-intermediates (Table 1). The modifications in the levels of these TCA cycle intermediates coincide with decreased malate dehydrogenase and isocitrate dehydrogenase activities (Figure 4). Moreover, we found that malate dehydrogenase and isocitrate  dehydrogenase are interacting partners of 14-3-3 chi, kappa, and psi (Figure 5), with putative 14-3-3 binding motifs. Another study identified a different form of isocitrate dehydrogenase isoform as a possible interacting partner of 14-3-3 proteins [49]. The modifications of metabolites involved in TCA cycle by 14-3-3s were due to alterations in the activities of several TCA metabolic enzymes. It is highly likely that the interactions of 14-3-3 proteins and two TCA key enzymes, malate dehydrogenase and isocitrate dehydrogenase, are the crucial factor controlling these enzyme activities. Considering these results, we conclude that 14-3-3 proteins regulate TCA cycle through protein-protein interaction with several enzymes of TCA cycle.

In addition to TCA cycle, our findings show that overexpression of 14-3-3 proteins deregulate the shikimate pathway, which plays a pivotal role in the production of precursors for aromatic compounds including aromatic amino acids in plants [50]. The activity of shikimate dehydrogenase was down-regulated in 14-3-3 ox lines (Figure 4), and the shikimate level was higher in 14-3-3 kappa-KO plants (Table 1). The decrease of tyrosine and phenylalanine in 14-3-3 ox plants (Table 1) also supports the notion that 14-3-3 proteins affect the shikimate pathway.

Plant metabolic processes are complicate, delicate and tightly linked reciprocally. Plants therefore need multi-functional players that can modulate multiple processes as well as the steps in each process and 14-3-3 proteins are one of the best candidates for this role. 14-3-3 proteins reversibly interact with selectively phosphorylated form of proteins and are involved in affecting targets to function in multiple ways such as confirmation change, scaffolding, and altering cellular location [2]. This is why 14-3-3 proteins have hundreds of target proteins and their interactions are found ubiquitously. In this study, we took established individual metabolic processes such as nitrogen metabolism, and aimed to uncover the ubiquitous roles 14-3-3 proteins play in the tightly linked metabolic processes. In 14-3-3 ox plants, reduction of starch levels may be due to decreased activity of Glucose-1-phosphate adenylyltransferase (AGPase). AGPase catalyzes the synthesis of ADP-Glc, the glucosyl donor used by starch synthases for starch biosynthesis [51], and regulates carbon storage in *Arabidopsis *[52]. AGPase is subjected to transcriptional regulation in diverse tissues and additional regulatory mechanisms at the posttranscriptional level [53]. The activities of starch metabolic enzymes are modulated by effector molecules which are often metabolic intermediates, or by posttranslational protein modification like phosphorylation [52]. Recent studies implicate that reversible protein phosphorylation play a critical role in the regulation of starch related enzymes such as AGPase [54,55]. Phosphorylated AGPase is possibly a target of 14-3-3 proteins and the binding can be a way to control its activity. From our study, we conclude that modification of AGPase activity is caused by drastic changes of carbon compounds in the 14-3-3 ox plants and the binding of 14-3-3 proteins with the phosphorylated form of the AGPase. As a consequence, 14-3-3 ox plants have greatly reduced levels of key metabolites in glycolysis leading to the decrease in carbohydrate supply to TCA cycle and shikimate pathway. Assimilation of ammonium to glutamine and glutamate is also negatively regulated in 14-3-3 ox plants, also suppressing supply to TCA cycle (Figure 6). In our study, we revealed that malate dehydrogenase and isocitrate dehydrogenase are direct targets of 14-3-3 proteins (Figure 5). This result suggests a mechanism in which 14-3-3 proteins bind and regulate key enzymes of TCA cycle through altering conformational change or scaffolding via protein-protein interaction. In addition, the decrease of glutamine and glutamate content due to ubiquitous interaction with 14-3-3 proteins and nitrate reductase and GS limit input to TCA cycle (Figure 6). With these results, we theorize that the ubiquitous interactions between 14-3-3 proteins and multiple metabolic enzymes restrict input to TCA cycle and shikimate pathway and consequently, TCA cycle itself is modulated by 14-3-3 protein via protein-protein interaction.

## Conclusions

Integration of metabolome data with a panel of enzyme assays proved to be a powerful tool to further our understanding of the function of 14-3-3 proteins in the regulation of primary metabolism in *Arabidopsis*. We confirmed that 14-3-3 proteins modulate activities of key enzymes of carbon and nitrogen metabolism and that these modifications were associated with drastic changes in the carbon/nitrogen balance in plants. In this study, we provide a novel functional link between 14-3-3 proteins and TCA cycle. The modification of the multiple metabolites involved in TCA cycle may have occurred due to the modification of enzyme activities of TCA cycle. Furthermore, our findings suggest that 14-3-3 proteins regulate TCA cycle through their interactions with two key enzymes of TCA cycle and that 14-3-3 proteins regulate the shikimate pathway and thus the production of aromatic compounds.

## Methods

### Plant materials and growth condition

Plants were grown on low salt media (LSM; 1.25 mM KNO_3_, 2 mM Ca(NO_3_)_2_, 0.75 mM MgSO_4_, 0.5 mM KH_2_PO_4_, 50 μM H_3_BO_3_, 10 μM MnCl, 2 μM ZnSO_4_, 1.5 μM CuSO_4_, 0.075 μM NH_4_Mo_7_O_24_, 74 μM Fe-EDTA, pH 5.7) with 1% sucrose and 0.6% Seakem agarose at 22°C with 16 h daylight at 150 μmol m^-2 ^s^-1 ^[56]. The all *Arabidopsis *plants used in this study have the same ecotype background, Col-0. Plants overexpressing 14-3-3 kappa, 14-3-3 chi and 14-3-3 psi and the knockout mutants of 14-3-3 genes were used as described [13]. For metabolomic profiling and enzyme activity analysis, three days after germination, plants were transferred onto new LSM plates and grown vertically. To reduce the effect by the position of plates in the growth chamber, plates were moved every two days. After two weeks, shoots and roots were harvested separately.

### Metabolite profiling and statistical analysis

Metabolite profiling using GC-TOF-MS was performed as described in [57]. Briefly, three of the harvested shoot or root samples were pooled as a replicate. Six replicates per line were used for metabolite profiling. A total of 5 mg fresh weight of the shoot and root samples were subjected to derivatization. An equivalent 6 μg of the derivatized samples were injected into the GC-MS instrument. The non-processed data obtained were pre-processed using the hierarchical multivariate curve resolution method [58].

SIMCA-P +12 software (Umetrics, Umeå, Sweden) were used for multivariate statistical analyses (i.e., PCA and OPLS-DA) and the R statistical environment http://cran.r-project.org for other statistical analyses such as the cross-contribution compensating multiple standard normalization (CCMN) and calculation of a false discovery rate (FDR). The PCA and OPLS-DA models were used to visualize the high-dimensional data and determine the metabolomic variation between the control (wild type) and the mutants (ox and/or KO). PCA was carried out to show how different variables (metabolites) change in relation to each other. OPLS-DA, which is as an extension of the supervised multivariate regression method PLS, was employed to remove some variation which was uncorrelated to class separation.

Outliers in the GC-MS data were identified using missing value robust PCA [59] and removed prior to further analysis. Metabolite abundance estimates were log transformed and scaled to unit-variance where applicable. Analytical bias was monitored via 11 internal, isotope-labeled standards and removed using the CCMN [60]. To validate OPLS-DA models, we applied analysis of variance of cross-validated predictive residuals (CV-ANOVA) in the SIMCA-P software [61].

Differentially abundant metabolites were identified using the LIMMA package [62]. Briefly, a linear model was fitted to each metabolite to compare the levels of wild type with levels in the mutants. Significant changes were declared for metabolites with a FDR level < 0.05 [63].

The day and night change of metabolites were analyzed as described [64,65]. Three of the harvested shoots were pooled as a replicate and six to eight replicates per genotype were analyzed. For day condition, long-day-grown (16 h light/8 h dark) plants were harvested 1 hour before offset of light and for night condition the plants were harvested 1 hour before onset of light. All data sets were analyzed for statistical differences compared to wild type by t-test using Prism 5 program (GraphicPad software, La Jolla, USA).

### Enzyme and metabolite assays

Chemicals were purchased as described in [66]. Fifteen of the harvested shoots were pooled as a replicate and six to eight replicates per genotype were analyzed. For enzyme measurements, aliquots of 20 mg frozen FW were extracted by vigorous mixing with extraction buffer [65]. 6-phosphofructokinase, citrate synthase, isocitrate dehydrogenase, and malate dehydrogenase were assayed as described in [65]. Ribulose-bisphosphate carboxylase was assayed as described in [67]. Triose-phosphate isomerase was assayed as described in [68]. Phosphoglucomutase was assayed as described in [69]. UTP-glucose-1-phosphate uridylyltransferase was assayed as described [70]. Fructose-bisphosphate aldolase was assayed by incubating crude extract or dihydroxyacetone phosphate standards for 20 min in a freshly prepared medium containing 0 or 5 mM fructose-1,6-Bisphosphate, 1 U ml^-1 ^triose-P isomerase, 2 U ml^-1^glycerol-3P dehydrogenase, 0.3 mM NAD^+^, 5 mM MgCl_2_, 1 mM EDTA, 0.05% Triton X 100, and 100 mm tricine buffer, pH 8.5. The reaction was stopped by addition of an equal volume of 0.5 M HCl. After incubation for 10 min at RT and neutralization with 0.5 M NaOH, the glycerol-3-phosphate produced was determined using the glycerol-3-phosphate/dihydroxyacetone phosphate-based cycling protocol described in [66]. All other enzymes assays in this study were performed as described in [66]. The statistical differences between each genotype and wild type were analyzed by t-test.

### Protein-protein interaction assays

The interaction between three 14-3-3 isoforms (14-3-3 chi, At4G09000; 14-3-3 kappa, At5G65430; 14-3-3 psi, At5G38480), and two malate dehydrogenases (At1G04410 and At5G43330) and an isocitrate dehydrogenase (At4G35650) were confirmed using GAL4-based Matchmaker yeast two-hybrid system (Clontech). Target proteins were cloned into pGADT7 (Clontech) with the GATEWAY cassette (Invitrogen) and then transformed into yeast strain AH109 using the lithium acetate-mediated method. The 14-3-3 proteins were cloned into pGBKT7 with the GATEWAY cassette (Invitrogen) and then transformed into Y187 and were confirmed that there were no autocatalytic activities. GFP and empty vectors were used as a negative control for protein-protein interaction [71]. Skip19 (At4G05460) and ASK2 (At5G42190) were used as a positive control for protein-protein interaction [72]. Yeast transformation and protein-protein interaction assays on selective media (Synthetic Dropout (SD)-Leu/-Trp/-His/-Ade and SD-Leu/-Trp+X-α-gal) were performed according to the manufacturer's instructions.

## Abbreviations

14-3-3 ox: 14-3-3 overexpression line; chi-KO: 14-3-3 chi knockout line; GC-TOF-MS: gas chromatography-time of flight-mass spectrometry; GS: glutamine synthetase; kappa-KO: 14-3-3 kappa knockout line; PCA: principal component analysis Pred comp: predictive component; psi-RNAi: 14-3-3 psi RNAi line; TCA cycle: tricarboxylic acid cycle.

## Authors' contributions

All authors read and approved the final manuscript. CD and RS designed research; CD, MK, RS, AM, HR and RS performed research; CD, KS, MS and RS analyzed data; CD and RS wrote the paper.

## Supplementary Material

Additional file 1 **Changes in metabolite profiles**. Click here for file

Additional file 2 **The list of enzymes that were measured in this study**. Enzyme activities were determined in 14-3-3 overexpression plants and wild type Col-0 plants (WT). The asterisk indicates significantly different compared to WT as determined by t-test (*, P < 0.05, n > 7). Click here for file
