# Supplementary figures and images for: Determining novel functions of Arabidopsis 14-3-3 proteins in central metabolic processes
Source: BMC Syst Biol. 2011 Nov 21;5:192. doi: 10.1186/1752-0509-5-192 (PMC3253775; doi:10.1186/1752-0509-5-192)

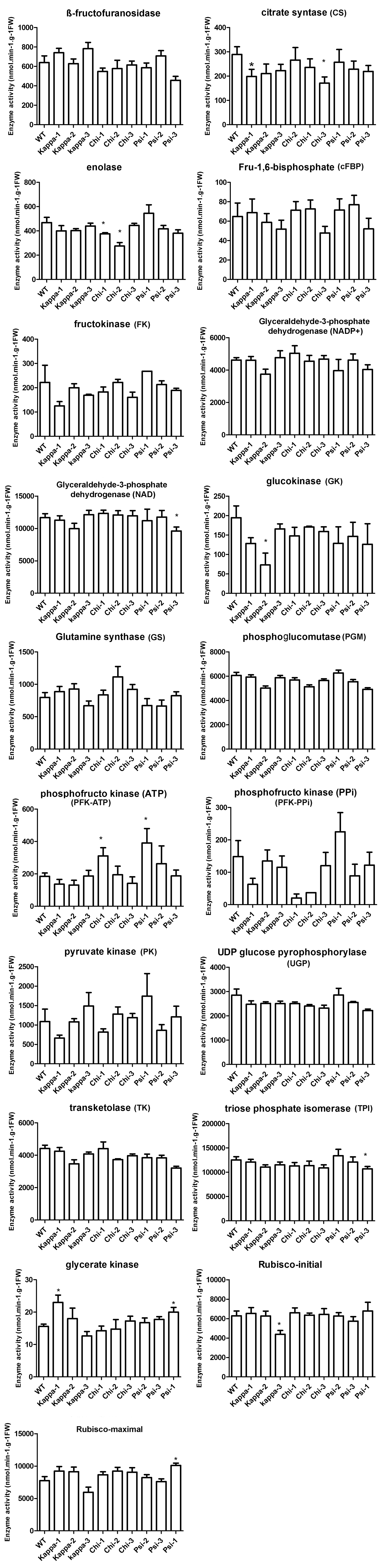

Supplement: Additional file 2 — The list of enzymes that were measured in this study. Enzyme activities were determined in 14-3-3 overexpression plants and wild type Col-0 plants (WT). The asterisk indicates significantly different compared to WT as determined by t-test (*, P < 0.05, n > 7). [file 1752-0509-5-192-S2.TIFF]
